# Toxicity Assessment of Resveratrol Liposomes-in-Hydrogel Delivery System by EpiVaginal^TM^ Tissue Model

**DOI:** 10.3390/pharmaceutics14061295

**Published:** 2022-06-17

**Authors:** May Wenche Jøraholmen, Pauliina Damdimopoulou, Ganesh Acharya, Nataša Škalko-Basnet

**Affiliations:** 1 Drug Transport and Delivery Research Group, Department of Pharmacy, Faculty of Health Sciences, UiT The Arctic University of Norway, Universitetsveien 57, 9037 Tromsø, Norway; natasa.skalko-basnet@uit.no; 2 Division of Obstetrics and Gynecology, Department of Clinical Science, Intervention and Technology, Karolinska Institutet and Karolinska University Hospital, SE-14186 Stockholm, Sweden; pauliina.damdimopoulou@ki.se (P.D.); ganesh.acharya@ki.se (G.A.); 3 Women’s Health and Perinatology Research Group, Department of Clinical Medicine, UiT The Arctic University of Norway and Department of Obstetrics and Gynecology, University Hospital of North Norway, Sykehusveien 38, 9019 Tromsø, Norway

**Keywords:** vaginal drug delivery, toxicity, EpiVaginal™ tissue model, irritation, resveratrol, liposomes, chitosan hydrogel

## Abstract

The natural polyphenol resveratrol (RES) has shown great potential as an antimicrobial, including against microbes associated with vaginal infections. To fully exploit the activities of RES, an all-natural ingredients formulation for RES delivery at vaginal site has been developed, namely liposomes loaded with RES, incorporated into a chitosan hydrogel as secondary vehicle. Although considered non-toxic and safe on their own, the compatibility of the final formulation must be evaluated for its biocompatibility and non-irritancy to the vaginal mucosa. As a preclinical safety assessment, the impact of RES formulation on the tissue viability, the effect on barrier function and cell monolayer integrity, and cytotoxicity were evaluated using the cell-based vaginal tissue model, the EpiVaginal™ tissue. RES liposomes-in-hydrogel formulations neither affected the mitochondrial activity, nor the integrity of the cell monolayer in RES concentration up to 60 µg/mL. Moreover, the barrier function was maintained to a greater extent by RES in formulation, emphasizing the benefits of the delivery system. Additionally, none of the tested formulations expressed an increase in lactate dehydrogenase activity compared to the non-treated tissues. The evaluation of the RES delivery system suggests that it is non-irritant and biocompatible with vaginal tissue in vitro in the RES concentrations considered as therapeutic.

## 1. Introduction

Common vaginal infections such as bacterial vaginosis and candidiasis still lack efficient and safe treatment options [1]. Moreover, the incidence of sexually transmitted infections (STIs) caused by microbes with a decreasing susceptibility to existing antibiotics is continuously on the rise [2]. An estimated 70% of all women will suffer from vaginal infections at least once during their lifetime, potentially risking serious consequences due to unsuccessful treatment or resistant and recurrent infections [3]. Localized therapy of vaginal infections can enable a high drug concentration at the infection site and offer limited systemic drug exposure with reduced side effects and an improved safety profile of the drug [2]. An increasing amount of research is focused on the topical therapy of infections, aiming for improved treatment, reduced side effects, and decreased risk for the development of antimicrobial resistance (AMR). In the search for novel antimicrobial compounds to fight the increase in AMR, several substances of natural origin show promise as alternatives to antibiotics.

Resveratrol (RES) is a naturally occurring substance with many attractive properties and potential health benefits. Studies on RES are increasingly reported, and with many clinical trials ongoing, the potential for clinical use is likely [4,5]. Its antioxidative [6,7,8], anti-inflammatory [4,7,9], and antimicrobial [4,10,11,12] activities make RES an interesting alternative as an active ingredient in pharmaceutical formulations targeting treatment of infection and inflammation. The effect against fungi, virus, and bacteria associated with vaginal infection is also established [10,12,13]. Due to poor solubility and limited stability and bioavailability, the optimal exploitation of RES activities is dependent on a suitable carrier. Liposomes are well-established nanocarriers suitable for the delivery of polyphenols [14,15,16,17]. The phospholipid-based nanovesicles are biocompatible and allow entrapment of compounds of different molecular weights and lipophilicity [2]. However, liposomal formulations are of liquid nature and demands appropriate modifications to prolong their residence time at vaginal site, such as coating with a mucoadhesive polymer or a secondary vehicle providing mucoadhesive properties [18].

Chitosan is also a material of natural origin that is gaining a lot of attention due to its many benefits as a constituent or as an active ingredient of pharmaceutical formulations [18]. Considering vaginal administration, chitosan is among the most promising materials due to its mucoadhesiveness, intrinsic antimicrobial properties, stability, and low toxicity [19]. In current study, we utilized chitosan as a building block in hydrogel, acting as a secondary vehicle to the RES-containing liposomes. The pH of chitosan hydrogels is similar to and compatible with the physiological acidic pH in a healthy vaginal environment [20]. The combination of the two delivery systems, liposomes enabling a high load of poorly soluble RES and increasing its stability in biological environment and chitosan hydrogel allowing a prolonged residence time at vaginal site, is a step further in reaching the optimized formulation for vaginal administration. Moreover, the intrinsic antimicrobial property of chitosan can intensify the therapeutic potential of the delivery system by acting in synergy with the active ingredient. Additionally, its ability to disrupt bacterial biofilms is a great benefit when targeting vaginal bacterial infection and inflammation [21]. Based on these qualities, particularly the mucoadhesive and antimicrobial properties [19], chitosan was chosen as a suitable polymer.

We previously developed and characterized a RES liposomes-in-hydrogel delivery system for the localized treatment of vaginal infections and proved its anti-inflammatory and anti-chlamydial effect [12]. The delivery system is composed of all natural ingredients that are all considered non-toxic and safe. However, the need to demonstrate the compatibility of systems developed for vaginal administration is continuously in demand, and the lack of toxicity studies considering prolonged release and retention on vaginal tissue is still insufficient for the final formulations and a drawback in many studies [1,22]. Thus, as a first stage, we evaluated the cytotoxicity of RES formulation in HeLa cell culture at the considered therapeutic RES concentration [12], and as a step forward to clinical evaluation, we tested the tissue toxicity and irritancy of the formulation. Biocompatibility and non-irritancy to the vaginal mucosa are major characteristics for novel vaginal delivery systems and particularly relevant for pregnant patients is the avoidance of systemic toxicity [20,22]. In vivo assessment of vaginal formulations demands large animal models; moreover, there is an expectation to replace and reduce the number of animals used in research [23,24]. There are available in vitro assays and in vitro or ex vivo models as alternative methods for in vivo irritation and toxicity assessments [24,25,26,27,28]. Hence, to assess the biocompatibility of RES formulation, its effect on tissue viability, barrier function and tissue damage were evaluated by the cell-based tissue model, the EpiVaginal™ tissue, which was validated for in vitro safety and irritation studies [29]. The in vitro model allows preclinical safety assessments while avoiding the use of animals and is shown to be a highly reproducible method suitable for assessing the irritation and cytotoxicity of vaginal delivery systems [29,30]. Our evaluations suggest that the developed RES liposomes-in-hydrogel delivery system is biocompatible, non-irritant, and non-toxic to the vaginal mucosa in vitro in RES concentrations considered appropriate for therapy of vaginal infections.

## 2. Materials and Methods

### 2.1. Materials

Lipoid S 100 (>94% phosphatidylcholine) was provided by Lipoid GmbH, Ludwigshafen, Germany. Chitosan (medium molecular weight hydramer HCMF (350–600 kDa), degree of deacetylation 70–95%) was provided by Chitinor, Tromsø, Norway. EpiVaginal™ Tissue Model (VEC-100) and MTT kit (MTT-100) was the product of MatTek Corporation, Ashland, MA, USA. CellTiter-Glo^®^ Luminescent Cell Viability Assay was purchased from Promega Corporation, Madison, WI, USA. HeLa human cervix epitheloid carcinoma cells (ECACC 93021013) was the product of Merck KGaA, Darmstadt, Germany. Resveratrol (RES: 3,5,4′-trihydroxy-trans-stilbene, purity ≥ 99%), lactic dehydrogenase based in vitro toxicology assay kit, gentamicin solution (10 mg/mL), penicillin, streptomycin, L-glutamine, acetic acid, glycerol, low glucose Dulbecco’s modified eagle medium (DMEM), Hank’s balanced salt solution, fetal bovine serum, non-essential amino acids, and HCL were the products of Sigma-Aldrich, Chemie GmbH, Steinheim, Germany. All chemicals were of analytical grade.

### 2.2. Liposomal Preparation

Liposomes were prepared using the thin film method previously described [7]. Lipid (Lipoid S 100, 200 mg) was dissolved in methanol and mixed with RES (10 mg) dissolved in ethanol. To remove solvents, evaporation (Büchi rotavapor R-124 with vacuum controller B-721, Büchi Vac^®^ V-500, Büchi Labortechnik, Flawil, Switzerland) at 50 mm Hg and 50 °C was applied for at least 3 h. The resulting lipid film was rehydrated in distilled water (10 mL) and liposomal suspension was stored in refrigerator (4–8 °C) overnight prior to further use. Plain liposomes (not containing RES) were prepared in similar manner, using only lipid (Lipoid S 100, 200 mg).

Vesicle size was reduced by stepwise extrusion through polycarbonate membranes (Nuclepore Track-Etch Membrane, Whatman House, Maidstone, UK), with pore size of 0.8, 0.4 and 0.2 µm and 0.1 µm and each step was repeated five times [7]. Liposomal suspension was stored in refrigerator for at least 6 h prior to further use or characterization.

### 2.3. Liposomal Characterization

Liposomal size distribution and polydispersity was determined by using photon correlation spectroscopy (Submicron particle sizer model 370, Nicomp, Santa Barbara, CA, USA). Samples were prepared in a laminar airflow bench to avoid interference from dust particles, as described earlier [31], and analyzed in the vesicle mode and intensity-weight distribution. Samples were measured in triplicates of a run time of 10 min.

Zeta potential was determined by Malvern Zetasizer Nano Z (Malvern, Oxford, UK). Measurement cells (DTS1060) were rinsed with ethanol and filtrated water (0.2 µm) prior to loading of the sample. Liposomal samples were diluted to achieve the proper count rate (typically 1:20) as previously described [32]. Samples were measured in triplicate at room temperature (25 °C).

Free RES was separated from liposomally entrapped RES by dialysis. RES liposomes (2 mL) in dialysis tubing (Mw cutoff: 12–14,000 Da, Medicell International Ltd., London, UK) were dialyzed against distilled water (500 mL) for 4 h at room temperature [7]. Aliquots of both liposomal sample and dialysis medium were diluted in methanol, transferred to a microtitre plate (Costar^®^UV 96-well plate with a UV-transparent flat bottom, Acrylic, Costar^®^, Corning, New York, NY, USA), and amount of RES was determined spectrophotometrically (Microtitre plate reader; Spectra Max 190 Microplate, Spectrophotometer Molecular devices, Sunnyvale, CA, USA) at 306 nm.

### 2.4. Liposome-in-Hydrogel Preparation

Chitosan hydrogel was prepared as previously described [12]. Briefly, medium molecular weight chitosan (350–600 kDa, DD 70–95%) was dispersed in a mixture of 1% (*w*/*w*) acetic acid and 10% (*w*/*w*) glycerol, to a final polymer concentration of 2.5% (*w*/*w*). The hydrogels were left to swell at room temperature (23–25 °C) for 48 h, forming a semi-solid formulation. Liposomes containing RES (free of unentrapped RES) were added to the chitosan hydrogel to a final concentration of 20% (*w*/*w*) liposomal suspension corresponding to 171–185 µg/g RES in formulation.

### 2.5. Cell toxicity Test

In vitro cell toxicity was evaluated by measuring the cellular ATP content using a luciferase-based assay (CellTiter-Glo^®^). HeLa cells were grown in low glucose Dulbecco’s modified eagle medium (DMEM) complimented with glutamine (1%), non-essential amino acids (1%), fetal bovine serum (10%), and antibiotics penicillin and streptomycin (2%). An appropriate number of cells were added to 96 well plates, and when confluent, media (100 µL) mixed with formulations corresponding to RES concentrations of 1.5, 3, 6, and 60 µg/mL were applied in direct contact with the cells. When exposed to the free RES, RES liposomes, and RES liposomes-in-hydrogel formulations for 48 h (incubation at 37 °C and 5% CO_2_), CellTiter-Glo^®^ reagent equal to the volume of cell culture medium (100 µL) was added to the wells. The plates were put on a shaker for 2 min to induce cell lysis followed by incubation at room temperature for 10 min to stabilize the luminescent signal. Luminecense was recorded using SpectraMax i3x Multi-Mode Microplate Reader (Molecular Devices, San Jose, CA, USA). A similar procedure was applied for the 120-h viability experiment, all except for a half media change (50 µL) after 48 h. Moreover, to obtain IC_50_, the same method was followed, applying media containing formulations corresponding to RES concentrations of 0.1, 1, 10, and 100 µg/mL. Cellular ATP was measured after exposure to formulation for 48 h.

### 2.6. EpiVaginal Tissue

Safety in human vaginal tissue was assessed using the EpiVaginal™ tissue model (MatTek VEC-100, MatTek Corporation, Ashland, MA, USA), a model that is validated for studies on vaginal irritation [29]. Dilutions of RES formulations were prepared in ultrapure water to desired RES concentrations of 1.5, 3, 6, and 60 µg/mL. Preparation of tissue models was done according to assay protocol, and they were incubated for 1 h (37 °C and 5% CO_2_) prior to dosing of test formulations in direct contact with tissues. Media was replaced by fresh media (900 µL) and diluted formulations (100 µL) and controls (negative; ultrapure water, positive; triton) were applied to the cell culture on top of the EpiVaginal™ tissue. Tissues were incubated for 24 h. Liquid on top of the tissues was decanted before the tissues were carefully rinsed twice to remove any residual sample. Trans epithelial electrical resistance (TEER) was measured (Section 2.6.2). All media was collected for lactate dehydrogenase (LDH) measurements (Section 2.6.3) and stored in a refrigerator.

#### 2.6.1. MTT Cytotoxicity Assay

Tissue viability as a function of mitochondrial activity was determined by MTT assay. After 24 h of exposure to the RES formulations, the tissues were placed in MTT solution (300 µL) and incubated for 3 h. Tissues were removed from MTT solution and rinsed before the inserts were immersed in extractant solution (2 mL). Plates were placed in a sealed bag and left on an orbital shaker for 2 h in room temperature and covered from light using aluminum foil. Extraction solution was transferred back in the respective wells and mixed before 200 µL was pipetted to a 96 well plate. Optical density was measured at 570 nm using SpectraMax i3x Multi-Mode Microplate Reader (Molecular Devices, San Jose, CA, USA) and measurements normalized as a percentage of non-exposed control tissues.

#### 2.6.2. Transepithelial Electrical Resistance (TEER)

The effect on barrier function of the EpiVaginal™ tissue model after exposure to the RES formulations was measured by TEER, using Millicell^®^ ERS-2 Voltohmmeter (EMD Millipore Corporation, Billerica, MA, USA). Resistance was measured after 24 h exposure to formulations and the measurements were done in duplicates and repeated, resulting in a total of four measurements for each insert. The surface area of the tissue (0.5 cm^2^) was taken into account and measurements were normalized as a percentage of the non-exposed control tissues.

#### 2.6.3. Lactate Dehydrogenase (LDH) Cytotoxicity Assay

The leakage of LDH enzyme from cells serves as an active biomarker for cellular or tissue damage. Hence, at the end of EpiVaginal™ tissue model experiment, after 24 h exposure to the RES formulations, the media was collected to measure the release or leakage of LDH into the culture medium. Aliquots of media (100 µL) were transferred to a 96 well plate and added LDH assay mixture (200 µL). The plate was covered from light using aluminum foil and incubated in room temperature for 30 min. The reaction was terminated by addition of 1 N HCL (30 µL). Absorbance was measured at 490 nm using SpectraMax i3x Multi-Mode Microplate Reader (Molecular Devices, San Jose, CA, USA).

### 2.7. Statistical Analyses

Statistical analysis was done by Student’s *t*-test to compare two means, and one-way ANOVA (analysis of variance) to compare > two means. A *p*-value less than 0.05 was considered statistically significant.

## 3. Results

### 3.1. Liposomal Characteristics

Plain liposomes and liposomes containing RES were characterized regarding the vesicle size, size distribution, and zeta potential. Moreover, RES entrapment efficiency was determined for liposomes containing RES (Table 1). The average vesicle size of both plain liposomes (155 ± 20 nm) and RES liposomes (155 ± 24 nm) was within the desired size range of 100–200 nm [12]. Liposomal suspensions expressed low polydispersity index (PI) with 0.077 and 0.079 for plain liposomes and RES liposomes, respectively, indicating a narrow size distribution. A spherical morphology and corresponding size for the RES liposomes have previously been confirmed by TEM images [12].

As expected, both plain and RES liposomes exhibited a close to neutral zeta potential with −0.96 ± 1.26 mV and −5.65 ± 1.82 mV, respectively. The entrapment efficiency was found to be high with 89% of RES in liposomes; this is in agreement with our earlier findings [7,12,33] and literature [6,34,35,36]. In previous studies, the RES release from the delivery system was thoroughly evaluated confirming a sustained RES release from both liposomal and liposomes-in-hydrogel formulations compared to the respective controls [12,33]. Moreover, the concentration of released RES was found to be above the effective concentrations required to induce desired anti-inflammatory and anti-chlamydial effect [12].

### 3.2. In Vitro Cell Toxicity

**Figure 1:**
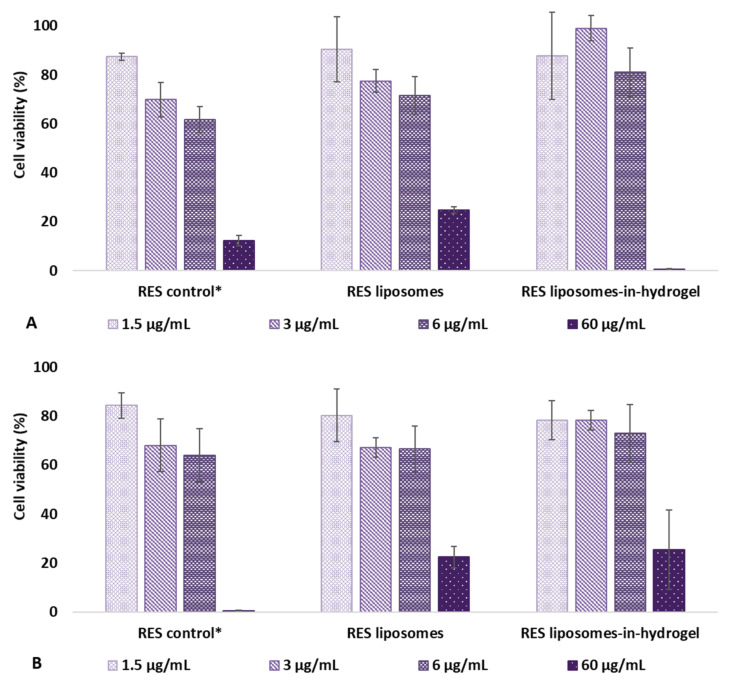
Cell viability after treatment for (**A**) 48 h and (**B**) 120 h (*n* = 3). Concentration of formulations is expressed as RES concentrations. * RES in propylene glycol.

The effect of RES formulations on cell toxicity was evaluated on HeLa cells using CellTiter-Glo^®^ reagent. The reagent results in cell lysis generating a luminescent signal corresponding to the amount of ATP present, and the amount of ATP is proportional with to the number of cells in the culture enabling a determination of viable cells. The cell viability was measured after treatment with RES in solution (control), RES liposomes, and RES liposomes-in-hydrogel. The results are expressed as percentage of viable cells when the cells were exposed to the formulations for 48 and 120 h, compared to non-treated cells (media only) and shown in Figure 1.

The highest RES concentration showed a significant effect on the cell viability for all formulations both after 48 h and 120 h of exposure. For the lower concentration (1.5 µg/mL), there was no significant difference in viability expressed by the formulations compared to the non-treated cells after 48 h. Moreover, no difference was seen between the tested formulations. However, at 3 µg/mL, the RES liposomes-in-hydrogel expressed a superior viability compared to the control with free RES (*p* < 0.0005). Moreover, among formulations with RES concentrations of 1.5, 3 and 6 µg/mL, RES liposomes-in-hydrogel 3 µg/mL was the only formulation that expressed a slight reduction in viability after 120 h compared to 48 h; however, the viability was still measured to be 78% (Figure 1).

In a separate experiment, the 50% viability (IC_50_) was measured after exposure to formulations with RES concentrations of 0.1–100 µg/mL for 48 h. RES liposomes were found to have a IC_50_ of 63 µg/mL, while for both RES in solution (control) and RES liposomes-in-hydrogel the viability was decreased by 50% at RES concentrations of 53 µg/mL and 52 µg/mL, respectively (*n* = 3). These results are reasonable compared to the earlier findings suggesting that RES liposomes-in-hydrogel formulations are not toxic after 24 h exposure in concentrations up to 50 µg/mL [33].

Plain liposomes (free of active ingredient) and plain hydrogel were also tested in liposomal or chitosan concentrations corresponding to RES containing formulations, to assess the influence of delivery system on its own (data not shown). The plain liposomes had a negligible impact on the cell viability at all concentrations after 48 h. After 120 h of exposure the plain liposomes expressed a cell viability corresponding to the RES liposomes, and the only variation was seen for the most concentrated liposomal sample that showed a 58% viability for plain liposomes compared to 26% for RES liposomes. No difference in cell viability was seen for plain hydrogel and RES liposomes-in-hydrogel.

### 3.3. Safety Evaluation on Cell-based Tissue Model

EpiVaginal™ tissue model has been validated for in vitro safety and irritation studies [29]. The cell-based tissue inserts were exposed to RES in solution (control), RES liposomes, and RES liposomes-in-hydrogel in the RES concentrations of 1.5, 3, 6, and 60 µg/mL to assess the safety of formulation for vaginal application. The tissue model enabled the evaluation of tissue viability as a function of mitochondrial activity, effect on barrier function, and cellular or tissue damage by LDH leakage after treatment with the RES formulations.

#### 3.3.1. MTT Tissue Viability Experiment

**Figure 2:**
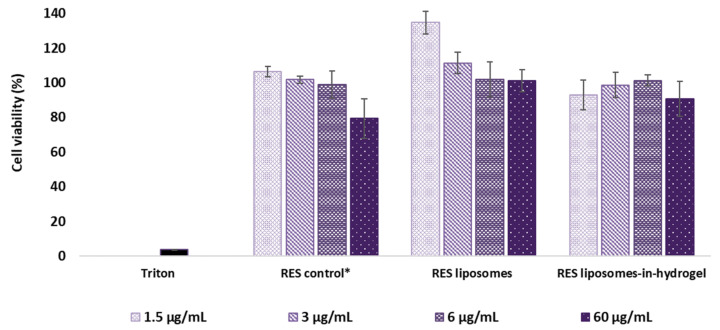
MTT cell viability after 24 h exposure (*n* = 3). Concentration of formulations is expressed as RES concentrations. * RES in propylene glycol.

The reduction of MTT has been shown to coincide with the cell viability [29] and was used to evaluate the effect of RES formulations on the tissue. The measurement was performed after 24 h and the results are expressed as percentage of viable cells after exposure to RES formulations compared to non-treated tissue inserts (ultrapure water). Hence, the higher percentage of cell viability means lesser toxic effects of the RES formulations and the results are shown in Figure 2. Triton was applied as a positive control.

RES liposomes and RES liposomes-in-hydrogel formulations exhibited no effect on cell toxicity in RES concentration up to 60 µg/mL compared to the viability in cells from non-treated tissues. In fact, liposomal RES expressed a superior cell viability (*p* ≤ 0.005) compared to the non-treated cells and cells treated with free RES (control) and RES liposomes-in-hydrogel (*p* ≤ 0.006) in lower concentrations 1.5 and 3 µg/mL (Figure 2). Plain chitosan hydrogel, free of liposomes and RES, was also applied to tissues in chitosan concentrations corresponding to the RES liposomes-in-hydrogel samples tested. The effect of plain hydrogel corresponded to the values found for the RES formulation (data not shown).

#### 3.3.2. Impact on Cell Monolayer Integrity

**Figure 3:**
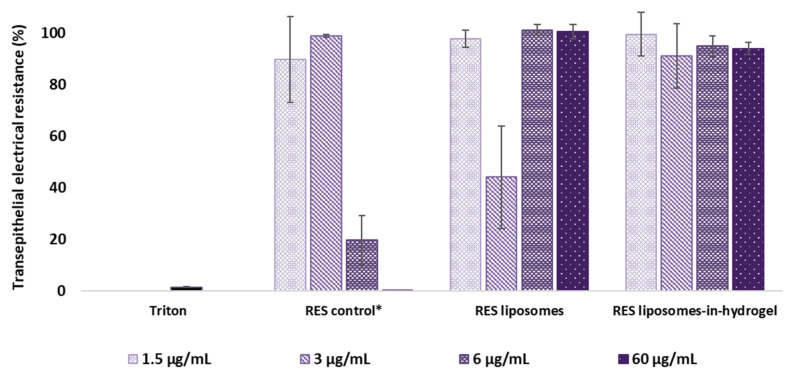
Transepithelial electrical resistance after 24 h exposure (*n* = 3). Concentration of formulations is expressed as RES concentrations. * RES in propylene glycol.

Transepithelial electrical resistance (TEER) enables measurements of the impact of drugs and formulations on cell monolayer integrity [37] and allows for quantitative evaluation of the barrier property of the entire tissue surface [30]. The results are expressed as percentage of electrical resistance in tissue after exposure to RES formulations compared to non-treated tissue inserts (ultrapure water) and shown in Figure 3. Triton was applied as a positive control.

Free RES in propylene glycol (control) made a substantial impact on the tissue integrity in RES concentration as low as 6 µg/mL and expressed similar damage as the positive control (triton) in the highest concentration (60 µg/mL). When RES was in liposomes or liposomes-in-hydrogel delivery system, the properties of the tissue were not affected compared to the non-exposed tissues. However, the impact of RES liposomes with RES concentration of 3 µg/mL clearly deviated from the anticipated result with a prominent reduction in transepithelial electrical resistance compared to both the higher concentrations and the other formulations. The impact of plain hydrogel was similar to that of the RES liposomes-in-hydrogel (data not shown).

#### 3.3.3. Lactate Dehydrogenase (LDH) Activity

**Figure 4:**
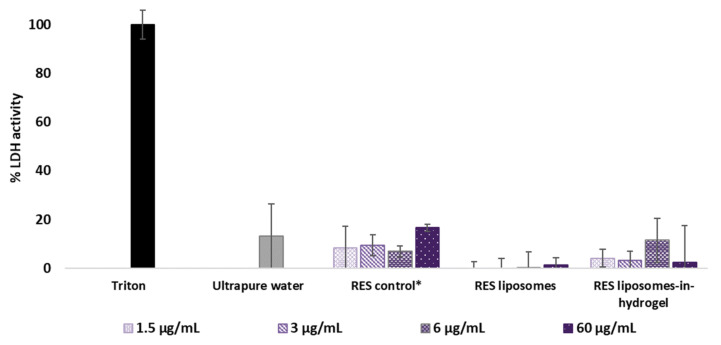
Lactate dehydrogenase activity after 24 h exposure (*n* = 3). Concentration of formulations is expressed as RES concentrations. * RES in propylene glycol.

Cellular or tissue damage leads to the leakage of LDH enzyme and the measurement of released LDH from dead or damaged cells can be directly related to cytotoxicity [38]. Released LDH generates pyruvate and NADH, which converts tetrazolium salt into formazan with intense red color, which can be measured spectrophotometrically. The amount of formazan is directly proportional to the amount of LDH in the cell-free supernatant collected from tissue inserts [39]. Based on a 100% cell lysis caused by the positive control (triton), the LDH activity of tissues exposed to RES formulations was compared to the activity caused by non-exposed (ultrapure water) tissues. Results are presented as percentage values relative to both positive and negative control [40] and shown in Figure 4.

None of the tested formulations expressed an increase in LDH activity compared to the non-treated tissues, and the results show no significant toxicity at any of the tested concentrations. LDH activity caused by plain chitosan hydrogel corresponded to the values found for the RES liposomes-in-hydrogel in the lower concentrations; however, the highest concentration showed LDH activity of 46% (data not shown) based on a 100% cell lysis caused by triton.

## 4. Discussion

The natural polyphenol RES has been shown to act as a potent antioxidant [7,9] and has demonstrated multiple beneficial effects, including as an antimicrobial [9,10,12] and anti-biofilm agent [41,42]. Hence, RES is a promising candidate as novel antimicrobial agent in the treatment of infections, including localized therapy of vaginal infections. RES has expressed efficacy against several microbes causing vaginal infection, such as herpes simplex virus [13] and *Chlamydia trachomatis* [12,43,44]. However, the poor solubility and low bioavailability of RES limit its clinical efficacy; thus, to benefit from the antimicrobial effects of RES when applied at vaginal site, a delivery system that can protect the substance from degradation and ensure a prolonged release and therapeutic effect is necessary. Liposomes have several attractive properties such as biocompatibility, ability to incorporate both hydrophilic and lipophilic substances, controlled release, targeting, and safety [45]. The use of liposomes to solubilize and entrap RES enables a high concentration of the active substance at the site of action as well as protection from vaginal environment. Chitosan hydrogel as a secondary vehicle provides the mucoadhesive properties necessary to ensure a sufficient residence time at vaginal site. Moreover, chitosan can strengthen the therapeutic potential of the system due to its intrinsic antimicrobial activity [18] and its ability to disrupt bacterial biofilms often associated with vaginal infection [21].

Compatibility with the vaginal environment is essential for all vaginal products. A successful local treatment of vaginal infections should not affect the normal vaginal physiology and microbiota. The recommended toxicity test for vaginal formulations is the rabbit vaginal irritation model; however, differences in anatomy and pH limit the reliability to human vaginal conditions [30]. Moreover, the strive to avoid the use of experimental animals emphasizes the importance and benefits of in vitro and ex vivo models [23,24]. RES, lipids in liposomes, and chitosan in hydrogel are all of natural origin and are confirmed to be non-toxic on their own; moreover, preliminary cytotoxic results suggest that RES liposomes-in-hydrogel formulations are not toxic to living cells in concentration up to 50 µg/mL [33]. Therefore, to further evaluate the safety of the formulation for vaginal application, we focused on the cell viability after the prolonged exposure and the impact of RES formulations on the cell-based vaginal tissue in vitro model, namely the EpiVaginal™ tissue model.

### 4.1. RES Formulation

Extensive characteristics of liposomal RES and RES liposomes-in-hydrogel have been evaluated in previous work. We have established a high RES load by liposomal entrapment [7,12,33] and optimized the RES liposomes-in-hydrogel formulation regarding the liposome-hydrogel ratio, texture, and mucoadhesive properties [12,33]. Moreover, the RES in vitro release from liposomes and liposomes-in-hydrogel formulation has been thoroughly evaluated [7,12,33], and a very limited RES penetration through sheep vaginal tissue ex vivo has been confirmed [33]. Consequently, the optimized formulation was used in this work to evaluate the toxicity.

RES has been shown to be safe in several clinical trials. However, bioavailability is not optimal [4,46]. The use of nanocarriers to improve the bioavailability and maintain or improve the safety of RES is continuously explored to pursue its translation into clinical use [47,48]. Liposomes are known to be biocompatible; additionally, numerous studies show that liposomal entrapment can reduce the toxicity of incorporated compounds [49,50]. The low toxicity of chitosan is also well established [51]. Hence, the delivery system comprising the natural ingredients RES, liposomes, and chitosan hydrogel, all considered non-toxic, should be safe. However, the biocompatibility and non-irritancy to the vaginal mucosa are major characteristics which also need to be assessed for the final formulation.

### 4.2. Cell Viability by Measurement of Cellular ATP

Cytotoxicity assays form a gold standard regarding in vitro safety evaluations of substances in cultured cells [52]. Luminescence-based cytotoxicity assays measure the cell death based on the amount of ATP present in living cells, as the reduced ATP level in cells indicates cell death. HeLa cells were exposed to the formulations of relevant RES concentrations for 48 or 120 h. RES concentrations of the tested formulations were the considered therapeutic concentrations, suggested to be up to 6 µg/mL based on earlier findings where RES formulations were challenged against *Chlamydia trachomatis* infection in vitro [12]. Even though the formulations are not expected to reside at the vaginal site for such a long period of time, this long-term exposure could provide an idea of the impact of repeated applications during the treatment one would administer. The result of the cytotoxic assay indicates that prolonged exposure does not significantly affect the viability of cells in vitro at therapeutic RES concentrations. All the tested formulations, except for RES liposomes-in-hydrogel 3 µg/mL, expressed a maintained cell viability after 120 h exposure compared to the respective samples after 48 h. However, the slight reduction was not significant, and the toxicity of the optimized formulation was found acceptable. A prominent cell toxicity was expressed in cultures exposed to RES concentrations of 60 µg/mL for 48 h. This was also confirmed by the 50% viability (IC_50_) evaluated in a separate experiment that showed that all tested formulations achieved 50% viability with a RES dose in the range of 52–63 µg/mL after 48 h treatment.

### 4.3. Preclinical Safety Assessment

The toxicological evaluations of pharmaceutical applications are often reliable in animal studies; however, the replacement of animals, reduction in number of animals used, and refinement of animal welfare in research is desirable [23]. Moreover, the in vivo testing of vaginal delivery systems demands large animal models that, nevertheless, lack correlation to the human vagina [30]. The complexity of the vaginal epithelium and the vagina as a site of drug delivery is a challenge in the development of vaginal formulations, and consequently also in the establishment of the adequate and standardized in vitro and ex vivo models [24]. There are several alternative models to in vivo testing, such as in vitro assays [25,26], in vitro models [27,29,30,52], and ex vivo models [28,52,53] for assessment of vaginal irritation and toxicity. The EpiVaginal™ tissue model is widely used for the in vitro safety and irritancy evaluations, and applied to study various vaginal applications, such as topical contraception [54], lubricants [30], antiviral nanoparticles [55,56], and topical microbicides [57,58], as well as studies of interactions between vaginal epithelium and bacteria [59] and as antiviral assay by introducing herpes simplex virus infection in EpiVaginal^TM^ tissue [60]. Moreover, the model can be applied as a cell-based tissue model to evaluate the in vitro drug permeability for vaginal drug delivery systems [29,61,62].

Several in vitro models based on vaginal cells are available for safety and permeability studies, but they do not include the mucus layer [63]. Due to the lack of mucus-producing cells in the cell-based vaginal tissue model used in this study, the impact of mucus is not accounted for in the safety evaluation. However, this would be essential primarily when evaluating the permeability of an active molecule using the in vitro model. This preclinical safety assessment enabled the evaluation of the impact of RES formulations on the tissue viability as a function of mitochondrial activity, the effect on barrier function and cell monolayer integrity, and cytotoxicity based on the leakage of LDH enzymes from damaged or dead cells.

#### 4.3.1. Tissue Viability Based on Mitochondrial Activity

The MTT assay is one of the most widely applied cytotoxicity assays [52]. The mitochondria of viable cells reduce MTT, a water-soluble tetrazolium salt, into the insoluble compound formazan. Formazan does not permeate the cell membrane, so it accumulates in healthy, metabolic viable cells [52,64,65].

Our findings showed that the RES formulations did not reduce the cell viability in the tissue model after treatment, and the RES liposomes showed a superior cell viability compared to the non-exposed tissues. This suggests that the liposomal entrapment of RES increased the cell viability compared to free RES. The reduced cytotoxic potential of drugs when in liposomal formulations has been presented for several active compounds as well as for liposomes with various lipid compositions [48,66]. Cell viability of the current liposomes-in-hydrogel formulation containing RES has previously been evaluated by MTT assay in HaCaT cell culture and expressed no effect on cell toxicity up to the concentration of 50 µg/mL [33]. This assay measuring the mitochondrial activity is considered to be one of the most sensitive in detecting cytotoxic events [65] and the obtained results are promising, indicating the safety of local administration of the RES liposome-in-hydrogel formulation.

#### 4.3.2. Tissue Viability Based on Cell Monolayer Integrity

Vaginal products should be compatible with the vaginal environment and not impact the epithelial barrier function. The vaginal mucosa is composed of a stratified squamous epithelium that forms an effective barrier to pathogens, and changes in the barrier function can impair this natural protective barrier, potentially resulting in inflammatory conditions and increased susceptibility to infection [67]. Changes in the barrier function can be evaluated by the presence of functional tight junctions. Epithelial layers in the EpiVaginal^TM^ tissue model have shown that they produce tight junctions and desmosomes between cells [29]. Tight junctions are responsible for the barrier function and can be evaluated by transepithelial electrical resistance (TEER) measurements quantifying the integrity of tight junctions in cell culture models [63,68].

TEER measurements of the tissue inserts exposed to the formulation confirmed that treatment with RES liposomes-in-hydrogel did not alter the integrity of the cell monolayer in RES concentrations up to 60 µg/mL. Moreover, the liposomal entrapment of RES clearly enhanced the RES compatibility with the tissues in the higher concentrations compared to the free substance in solution (RES control). This positive effect was maintained by the liposomes-in-hydrogel delivery system. However, RES liposomes with RES concentration of 3 µg/mL exhibited a prominent impact on the barrier function of tissue model. This clearly deviated from the anticipated result and did not coincide with the impact measured for the higher RES concentrations as well as the other formulations. The result might appear as a defective measurement, however, was seen for all three parallels. The 3 µg/mL RES liposomes did not express cell toxicity or detriment to the cell-based tissue model in any of the other evaluations.

The use of mucoadhesive polymers, such as chitosan, can enable the formulation to be in close contact with the vaginal epithelium. Due to disruption of intercellular junctions, chitosan can improve the bioavailability of the active ingredient by increasing epithelium permeability [3]. However, the information on biocompatibility of chitosan after application on vaginal mucosa is insufficient [1]. In addition to the RES formulations, plain chitosan hydrogel was tested on the cell-based tissue model, confirming the hydrogel did not alter the integrity of the cell monolayer. It is also worth mentioning that the presence of the mucus layer lining the vaginal epithelium influences the robustness of the epithelium [63] as the in vitro tissue model lack the natural mucus layer. Moreover, when evaluating the vaginal formulations for treatment of vaginal infections associated with mucosal lesions, it must be considered that the vaginal mucosa is already damaged [20].

#### 4.3.3. Tissue Viability Based on Cell Membrane Leakage

Lactate dehydrogenase (LDH) is a cytosolic enzyme that is released from cells due to plasma membrane leakage, demonstrating cell toxicity, and the LDH assay has been validated as an assessment of overall cell death [69]. Plasma membrane leakage causing the release of LDH enzyme is regarded as an early event in necrosis and a late event in apoptosis, hence, the lack of an increase in the LDH activity in the extracellular medium is not a definite proof that there is no toxicity. In general, no single toxicity assay can determine the type and extent of damage to the cells on its own [38]. The LDH release assays is a complementary test to the MTT reduction assay to evaluate cytotoxicity by two different mechanisms [70]. While the LDH leakage assay is based on the enzyme release into the culture medium after cell membrane damage indicating irreversible cell death, the MTT assay is based on the enzymatic conversion of MTT in the mitochondria [65].

Our findings that treatment with RES liposomes-in-hydrogel does not cause the LDH leakage from cells support the reported mitochondrial activity (MTT) and suggest that the RES formulations are non-toxic to the vaginal tissue up to a RES concentration of 60 µg/mL. The lack of mucus layer in the tissue model precludes a definite conclusion that the current formulations do not inflict any in vivo irritation; however, the accordant results suggest that the RES liposomes-in-hydrogel delivery system should not induce irritancy or toxicity to vaginal tissue and cells.

Although it is known that chitosan has intrinsic antimicrobial effects, the exact mechanisms of actions of chitosan are not yet established [18,19]. It is proposed that chitosan interacts with the negatively charged microbial cell membranes and causes disruption of the membrane and a leakage of intracellular constituents. Despite the proven toxic effects on microbial cells, chitosan is considered as a non-toxic, biocompatible material, and regarded as safe for pharmaceutical and biomedical application [71]. Although the plain chitosan hydrogel, free of RES liposomes, caused increased LDH leakage in the highest concentration, no effect was seen in the lower concentrations. Moreover, no leakage was seen for the final formulation, and our evaluation of RES liposomes-in-hydrogel delivery system confirm that it is non-irritant and biocompatible with the cell-based vaginal tissue model.

The findings from EpiVaginal^TM^ tissue model confirmed the safety of our formulation; however, there is a clear need for widely available, robust, and validated in vitro models that would permit the direct comparison of different formulations, particularly during preformulation development to facilitate optimization of vaginal formulations prior to in vivo challenge.

## 5. Conclusions

A liposome-in-hydrogel formulation was prepared with a high load of active ingredient, the natural occurring polyphenol RES. The safety of the formulation was evaluated by cell viability studies and preclinical safety assessments on the cell-based tissue model, the EpiVaginal^TM^ tissue, including complementary tests to demonstrate the biocompatibility of formulation with vaginal tissue. The novel system did not exhibit any in vitro toxic effect to live cells at considerable treatment concentration. All tested concentrations (up to 60 µg/mL) confirmed compatibility of tested formulations with the vaginal tissue model. Tissue integrity remained intact after the treatment with the RES liposomes-in-hydrogel formulation and no irritancy was detected. Moreover, none of the tested formulations caused cell membrane leakage at the tested concentrations. This strongly suggests that the system is compatible with vaginal tissue and can be considered safe for vaginal administration. However, it should be finally confirmed in in vivo studies.

## Figures and Tables

**Table 1:** Liposomal characteristics. Results are expressed as mean ± SD (*n* = 3).

Formulation	Vesicle Size (nm)	PI *	Zeta Potential (mV)	Entrapment Efficiency (%)
Plain liposomes	155 ± 20	0.077	−0.96 ± 1.26	-
RES liposomes	155 ± 24	0.079	−5.65 ± 1.82	89 ± 3

* Polydispersity index.

## Data Availability

Not applicable.
